# Combined genomic and structural analyses of a cultured magnetotactic bacterium reveals its niche adaptation to a dynamic environment

**DOI:** 10.1186/s12864-016-3064-9

**Published:** 2016-10-25

**Authors:** Ana Carolina Vieira Araujo, Viviana Morillo, Jefferson Cypriano, Lia Cardoso Rocha Saraiva Teixeira, Pedro Leão, Sidcley Lyra, Luiz Gonzaga de Almeida, Dennis A. Bazylinski, Ana Tereza Ribeiro de Vasconcellos, Fernanda Abreu, Ulysses Lins

**Affiliations:** 1 Instituto de Microbiologia Paulo de Góes, Universidade Federal do Rio de Janeiro, 21941-902 Rio de Janeiro, RJ Brazil; 2 School of Life Sciences, University of Nevada at Las Vegas, Las Vegas, NV 89154-4004 USA; 3 Departamento de Matemática Aplicada e Computacional, Laboratório Nacional de Computação Científica, 25651-070 Petrópolis, RJ Brazil; 4 Current institution: Departamento de Biologia, Universidade Federal de São Carlos, 18052-780 Sorocaba, SP Brazil

**Keywords:** Magnetotactic bacteria, Magnetosome, *Magnetofaba australis* strain IT-1, Biomineralization, *mam* genes, Genome

## Abstract

**Background:**

Magnetotactic bacteria (MTB) are a unique group of prokaryotes that have a potentially high impact on global geochemical cycling of significant primary elements because of their metabolic plasticity and the ability to biomineralize iron-rich magnetic particles called magnetosomes. Understanding the genetic composition of the few cultivated MTB along with the unique morphological features of this group of bacteria may provide an important framework for discerning their potential biogeochemical roles in natural environments.

**Results:**

Genomic and ultrastructural analyses were combined to characterize the cultivated magnetotactic coccus *Magnetofaba australis* strain IT-1. Cells of this species synthesize a single chain of elongated, cuboctahedral magnetite (Fe_3_O_4_) magnetosomes that cause them to align along magnetic field lines while they swim being propelled by two bundles of flagella at velocities up to 300 μm s^−1^. High-speed microscopy imaging showed the cells move in a straight line rather than in the helical trajectory described for other magnetotactic cocci. Specific genes within the genome of *Mf. australis* strain IT-1 suggest the strain is capable of nitrogen fixation, sulfur reduction and oxidation, synthesis of intracellular polyphosphate granules and transporting iron with low and high affinity. *Mf. australis* strain IT-1 and *Magnetococcus marinus* strain MC-1 are closely related phylogenetically although similarity values between their homologous proteins are not very high.

**Conclusion:**

*Mf. australis* strain IT-1 inhabits a constantly changing environment and its complete genome sequence reveals a great metabolic plasticity to deal with these changes. Aside from its chemoautotrophic and chemoheterotrophic metabolism, genomic data indicate the cells are capable of nitrogen fixation, possess high and low affinity iron transporters, and might be capable of reducing and oxidizing a number of sulfur compounds. The relatively large number of genes encoding transporters as well as chemotaxis receptors in the genome of *Mf. australis* strain IT-1 combined with its rapid swimming velocities, indicate that cells respond rapidly to environmental changes.

**Electronic supplementary material:**

The online version of this article (doi:10.1186/s12864-016-3064-9) contains supplementary material, which is available to authorized users.

## Background

Magnetotactic bacteria (MTB) comprise a morphologically and phylogenetically diverse group of ubiquitous, flagellated, aquatic bacteria capable of orienting along magnetic field lines because of the presence of nano-sized intracellular magnetic organelles called magnetosomes [1]. Each magnetosome consists of a nano-sized, membrane-bounded crystal of magnetite (Fe_3_O_4_) or greigite (Fe_3_S_4_) usually arranged as (a) chain(s) inside the cell. Magnetotactic cocci have continually been found to be the most abundant morphotype of MTB in freshwater, brackish and marine sediments [2]. While the phenotypic characterization of various magnetococci has been reported, information regarding their ecological, biogeochemical roles in natural environments is limited [3, 4]. In the magnetotactic cocci, as in other MTB, the magnetotactic response is modulated by chemotaxis (e.g., aerotaxis) in a phenomenon described as magneto-aerotaxis [5]. The magnetic alignment of the cells along the Earth’s geomagnetic field lines together with a strong chemotactic response maximizes the efficiency of the organism’s search for nutrients and energy (i.e., an optimal position in vertical chemical gradients in the environment) for survival and growth [1].

Genomic data are available for of a number of MTB (Additional file 1). Complete genomes have been described for some *Alphaproteobacteria* including *Magnetospirillum gryphiswaldense* strain MSR-1, *Magnetospirillum magneticum* strain AMB-1, *Magnetospirillum caucaseum* strain SO-1, *Magnetospirillum* sp. strain MS-1, *Magnetococcus marinus* strain MC-1 and *Magnetospira* sp. strain QH-2 [6–11]. For the *Deltaproteobacteria*, complete genome sequences are available for *Desulfovibrio magneticus* strain RS-1 [12]. Partial genomic information is also available for other cultured MTB affiliated with the *Alphaproteobacteria* [13, 14] and cultured and uncultured MTB belonging to the *Deltaproteobacteria* [15–18], *Nitrospirae* [19, 20], *Candidatus* Omnitrophica (part of the *Planctomycetes-Verrucomicrobia-Chlamydiae* (PVC) bacterial superphylum) [19] and possibly to the *Latescibacteria* [21, 22]. These partial genomic sequences include descriptions of *mam* genes for characterization of biomineralization processes in MTB except for the Latescibacterial SCGC AAA252-B13 genome which was used for the identification of an uncultured environmental microorganism [21] and for the detection of *mam* gene homology without characterization of morphological features of this putative MTB [22].

Magnetotaxis in MTB from both Hemispheres has been characterized [1]. In the Northern hemisphere, MTB predominantly swim northward (i.e., North-seeking) while in the Southern hemisphere, the majority of MTB tend to migrate southward (South-seeking). In both situations, the swimming direction is downward presumably to avoid toxic levels of O_2_ in surface waters and to find and maintain an optimal position in vertical chemical gradients [23]. Recently, a South-seeking strain named *Magnetofaba australis* strain IT-1 was isolated in pure culture [13]. *Mf. australis* strain IT-1 is a magnetotactic coccus that biomineralizes elongated cuboctahedral magnetite magnetosomes [13]. Here, we used a comprehensive approach combining both genomic and ultrastructural analyses to characterize its morphology and its potential roles in nitrogen, sulfur, iron and phosphate biogeochemical cycling.

## Results and discussion

### General genomic description

The draft genome of *Mf. australis* strain IT-1 has a size of 4,986,701 bp, with a coding density of 82.64 % represented by 4,130 loci, with an average length of 1,010 bp. A total of 2,886 loci encode proteins with putative functions, 44 encode tRNAs and 1,194 encode hypothetical proteins (Additional file 2). Although there is no evidence for the presence of extrachromosomal elements such as plasmids, the occurrence of several lengthy repeat regions hindered genome closure. The genome assembly contains 21 contigs, with a coverage of 33X. Gaps between contigs are mostly regions with unresolved repetitions, caused by the high number of transposable elements found in the IT-1 genome. The G + C content is 57.95 %, slightly higher than G + C content of *Mc. marinus* strain MC-1 (54.17 %) and lower than G + C values found in the freshwater magnetotactic spirillar strains *Ms. magneticum* strain AMB-1 and *Ms. gryphiswaldense* strain MSR-1 (65.09 and 63.28 %, respectively).

According to KEGG functional classification, most predicted ORFs are related to carbohydrate and amino acid metabolism (162 and 165 ORFs, respectively). A high number of ORFs are related to signal transduction, cell motility and membrane transport (156, 132 and 110 ORFs, respectively). As *Mc. marinus* strain MC-1 is the only magnetotactic coccus with an available complete genomic sequence, it is not surprising that 51.1 % of *Mf. australis* strain IT-1 predicted ORFs were more similar to ORFs described in *Mc. marinus* strain MC-1 than any other MTB. Nevertheless, similarity between many of their homologous gene sequences is relatively low. Other ORFs were more similar to *Magnetospirillum magneticum* (1.1 %) and to non-magnetotactic members of the *Proteobacteria* phylum (<1 % ORFs similar to various strains).

### Phenotypical characterization of *Mf. australis* strain IT-1

#### Ultrastructure and granular inclusions

**Fig. 1:**
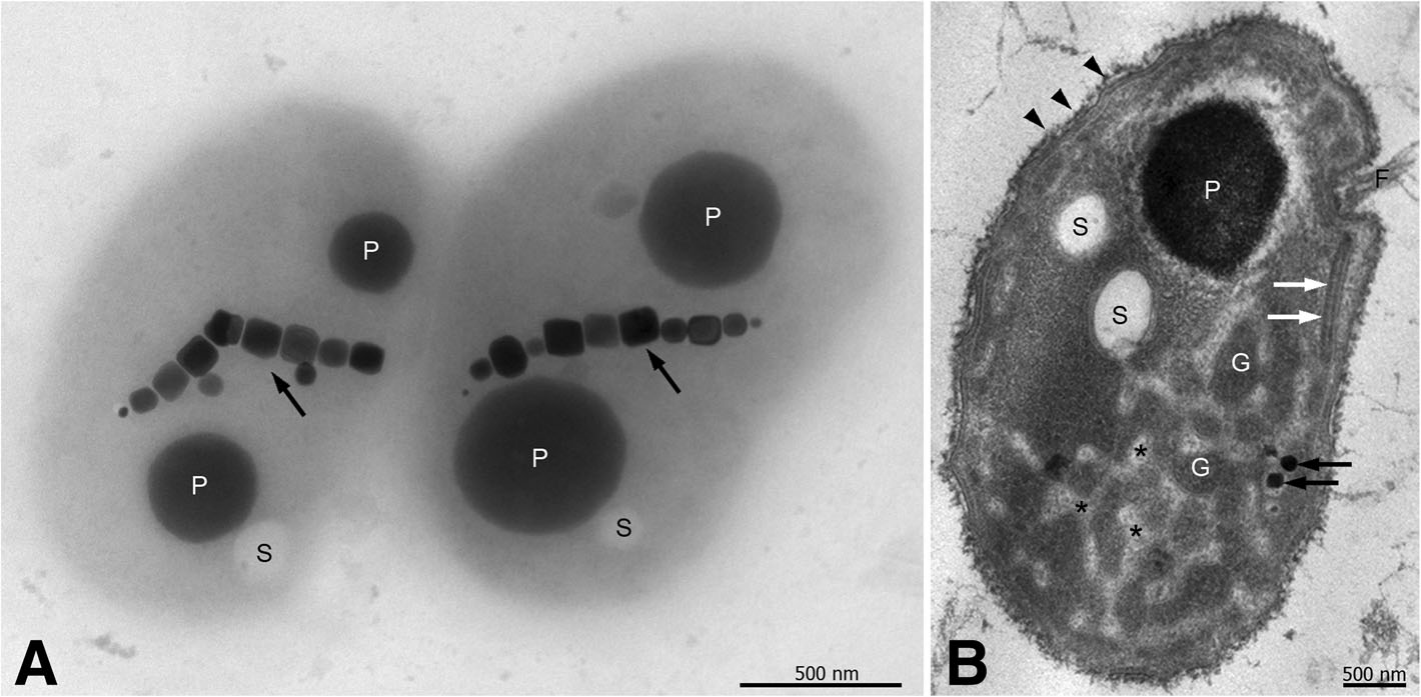
Ultrastructure of *Mf. australis* strain IT-1. Ultrastructure of *Mf. australis* strain IT-1. **a** Whole mount TEM image showing a single magnetosome chain, P-rich (P) and sulfur (S) granules; (**b**) Ultrathin section TEM image of high pressure frozen and freeze-substituted cells showing P-rich (P) and sulfur (S) granules, two magnetosomes (*black arrows*), a flagella bundle (F) associated with chemoreceptor array (*white arrows*), and a fibrillar layer at the cell surface (*arrowheads*). Uncharacterized globular structures (G) embedded in an electron-lucent material (asterisks) can be observed

Whole-mount transmission electron microscopy (TEM) of cells of *Mf. australis* strain IT-1 shows that they have a unique morphology having both a concave and convex surface (Fig. 1a) confirming the “faba” bean morphology of the cells. Each cell contains a single chain of elongated octahedral magnetosomes and intracellular granules described previously [13]. TEM of ultra-thin sections of cells (Fig. 1b) show that the overall cell ultrastructure of *Mf. australis* strain IT-1 is consistent with a two-membrane structure typical of Gram-negative bacteria with a turgid periplasmic gel between them. On the surface of the outer membrane a layer of fibrillar material is present and may represent some type of capsule or S layer (Fig. 1b; arrowheads); a similar structure was reported in uncultured magnetotactic cocci from the Itaipu lagoon, Brazil [24]. The cytoplasm contains a series of uncharacterized structured regions consisting of “pockets” of amorphous, globular electron-dense material interlaced with electron-lucent regions (Fig. 1b; asterisks). We observed a tabular periodic structure parallel to the inner membrane of some cells in close association with the flagella bundles, most likely corresponding to chemoreceptor arrays (Fig. 1b; white arrows), described also in *Ms. gryphiswaldense* strain MSR-1 [25], *Magnetovibrio blakemorei* strain MV-1 [26] and other motile non-magnetotactic strains from different bacterial phyla [27].

#### Flagellar apparatus and motility

**Fig. 2:**
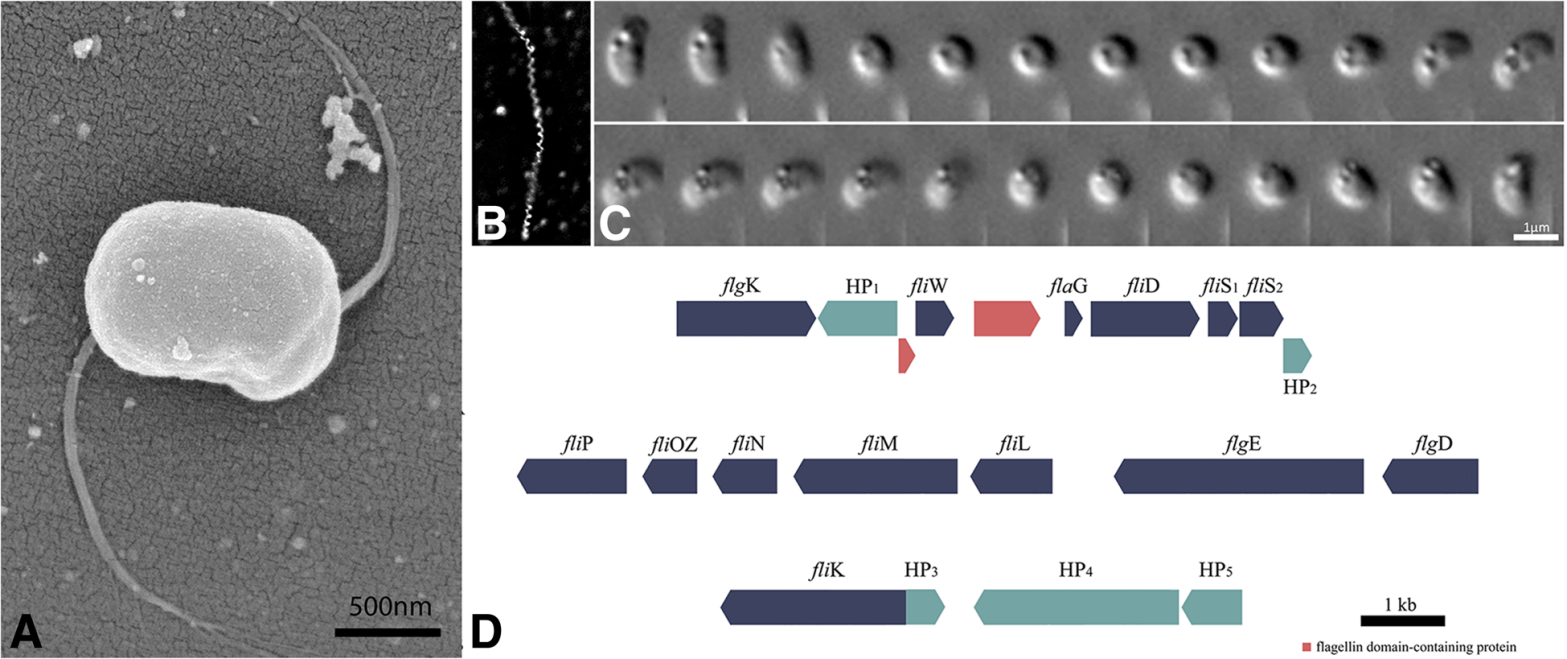
Flagellar apparatus and motility in *Mf. australis* strain IT-1. Flagellar apparatus and motility in *Mf. australis* strain IT-1. **a** Scanning electron microscopy of a cell with two flagella bundles; (**b**) Dark-field image recorded trajectory of a cell showing an “helical” path recorded for 1s; (**c**) Sequential series of light microscopy DIC images of a swimming (left to right, top to bottom) cell imaged with a high-speed camera. Each frame represents 1/1000s. The morphology of the cell is similar to a “faba” bean with a convex (*left*) and a concave (*right*) side; several granules can be seen in the cell body. **d** Organization of genes involved in flagellar apparatus biosynthesis in *Mf. australis* IT-1. Fourteen copies of *fliC* genes occur elsewhere in the genome. HP (*light blue*) are hypothetical proteins

Scanning electron microscopy (SEM) shows that cells possess two bundles of lophotrichous flagella, each at one extremity of the cell (Fig. 2a). These bundles propel cells at swimming speeds up to 300 μm s^−1^. Each flagella bundle consists up to seven separate flagella filaments, with a diameter of 13 ± 2 nm (*n* = 50) and a length of 1.9 ± 0.5 μm (*n* = 20). The flagella originate from within a pit located on the cell surface (Fig. 1b). In hanging drop assays under oxic conditions, *Mf. australis* strain IT-1 exhibited South-seeking polar magnetotaxis swimming in the presence of the magnetic field of a bar magnet with a fast back and forth swimming pattern near the edge of the drop. A helical trajectory was observed when movement was recorded with a CCD camera using dark-field microscopy (Fig. 2b). However, when analyzed using a high speed camera (500 to 1000 fps), we observed that over 90 % of *Mf. australis* strain IT-1 cells swim in a straight trajectory by rotating the cell body along an apparent axis parallel to the movement direction (Fig. 2c). This axis is inclined relative to the position of the magnetosome chain which lies along long axis of the cell. The flagella bundles occur at the concave surface of the cell. Surprisingly, *Mf. australis* strain IT-1 seems to swim with the concave surface forward (Fig. 2c), which strongly suggests that the flagella bundles rotate in front of the cell as the cell body moves.

In the genome of *Mf. australis* strain IT-1, genes encoding for every component of the flagellar apparatus (*flg*, *flh*, *fli*; Fig. 2d) are present and those for chemotaxis (*cheA*, *cheB*, *cheB*/*cheR*, *cheW*, *motA*, MCP to signal translation were also identified. There are approximately 64 genes involved in the synthesis of proteins related to flagellar apparatus and motility. FliPOZNMLWSQREFGI, FlgEKBCG, FlhAB, FlaG, FlgM and 14 copies of a protein containing a flagellar domain, FliC type (ORFs 1035, 1042 to 1044, 1047, 1049, 1059, 2652, 4643 and 5226 to 5230) were detected. These genes form separate groups along the genome (Fig. 2c). Interestingly, the arrangement of the contig containing *flgK*, *fliW*, *fliD*, *fliS*_*1*_ and *fliS*_*2*_ was similar to the closely related strain MO-1 [28], which is also capable of very high swimming speeds. Flagellin genes share similarity with *fliC* genes from magnetococcal strains MC-1 and MO-1, with a slightly higher (1-5 %) similarity with genes from the fast swimming MO-1.

Under aerobic conditions, as previously stated, over 90 % of the cells’ motility in *Mf. australis* strain IT-1 occur in linear rather than helical trajectories and at higher speeds (up to 300 μm.s^−1^, with average of 186 ± 63 μm s^−1^ [13]) than most MTB (average speed ranging from 10 to 120 μm s^−1^) [29, 30] and similar to that reported in strain MO-1 [31]. The linear trajectory and the magnetosome chain position in the cell raises important issues regarding magnetotaxis as an efficient mechanism for navigation. The magnetosome chain in *Mf. australis* strain IT-1 is positioned perpendicular to the axis of movement. Usually, the magnetosome chain is aligned with the axis of cell movement by flagellar propulsion which results in an efficient orientation along the magnetic field [23]. Although magnetosome chains are approximately perpendicular to the swimming axis in *Mf. australis* strain IT-1, cells orient along magnetic field lines and respond to changes in the magnetic field. Cells of the magnetotactic coccus strain MO-1 also swim in a straight trajectory, with the magnetosome chain not aligned to the axis of motility [32]. The lack of perfect alignment between the magnetosome chain and the magnetic field lines might be useful for cells to overcome obstacles in their trajectory. The mechanisms for magnetic field orientation and cell dislocation in vertical gradients by flagella propulsion differs from the traditional magnetotaxis model described for other MTB [5, 30]. It is possible that *Mf. autralis* strain IT-1 cells always swim with their flagella in front of the cell body and reversal of swimming direction is achieved by reversing the sense of flagella rotation, as described in other MTB strains [30]. Possibly, MTB described as moving along helical paths is a misinterpretation of results generated by a low speed imaging systems. Interestingly, a flagellar sheath, observed in other magnetotactic cocci or ovoid cells [33], was not observed in *Mf. australis* strain IT-1. Genes similar to that encoding the Sap protein [34], related to the flagellar bundle sheath in both MO-1 and MC-1 [10], were not found in the IT-1 genome, confirming our microscopy observations and implying that at least for strain IT-1, the sheath is not required for “smooth” swimming as has been suggested [34]. Uncultured cocci from the Itaipu lagoon, when analyzed by freeze-fracture, also did not present a sheath around its bundle of flagella [35]. On the other hand, some cocci showed an intricate arrangement of fibrils that may help to coordinate flagellar movement.

The chemoreceptor arrays, in close association with the flagellar bundles, might allow the cell to control and synchronize the direction and frequency of the rotation of the flagella in the bundle, obviating the need for the flagella sheath that works not only as a protection mechanism but also as a flagella rotation coordinator [34]. The proximity of the chemoreceptor array to the flagellar bundle might allow the cell to respond quickly to environmental changes with greater propulsive force necessary for fast swimming, ensuring cell survival. An MCP-like protein was shown to interact with MamK filaments in *Ms. magneticum* strain AMB-1 [36], but the similarity values between this protein and the ORFs annotated as MCP in *Mf. australis* strain IT-1 genome were not high enough to assign this function with certainty. However, due to the high plasticity of sensory domains, it is possible that a MCP whose ligand-binding domain is not described yet carries the coupling between chemotactic sensor and the magnetosome chain in *Mf. australis* strain IT-1. A very efficient locomotion system might have evolved in magnetotactic cocci that allowed them to move at high speeds to niches with suitable chemical gradients for their survival. This would make easier to respond to sudden changes in the vertical gradients that may occur in aquatic environments. With the exception of the marine *Magnetospira* sp. strain QH-2 [11], magnetotactic species with their genome sequenced have a large number of MCP-related genes, characteristic of motile bacteria, with metabolic versatility and that occupies dynamic environments [37].

#### Magnetosome crystalline habit and genes

**Fig. 3:**
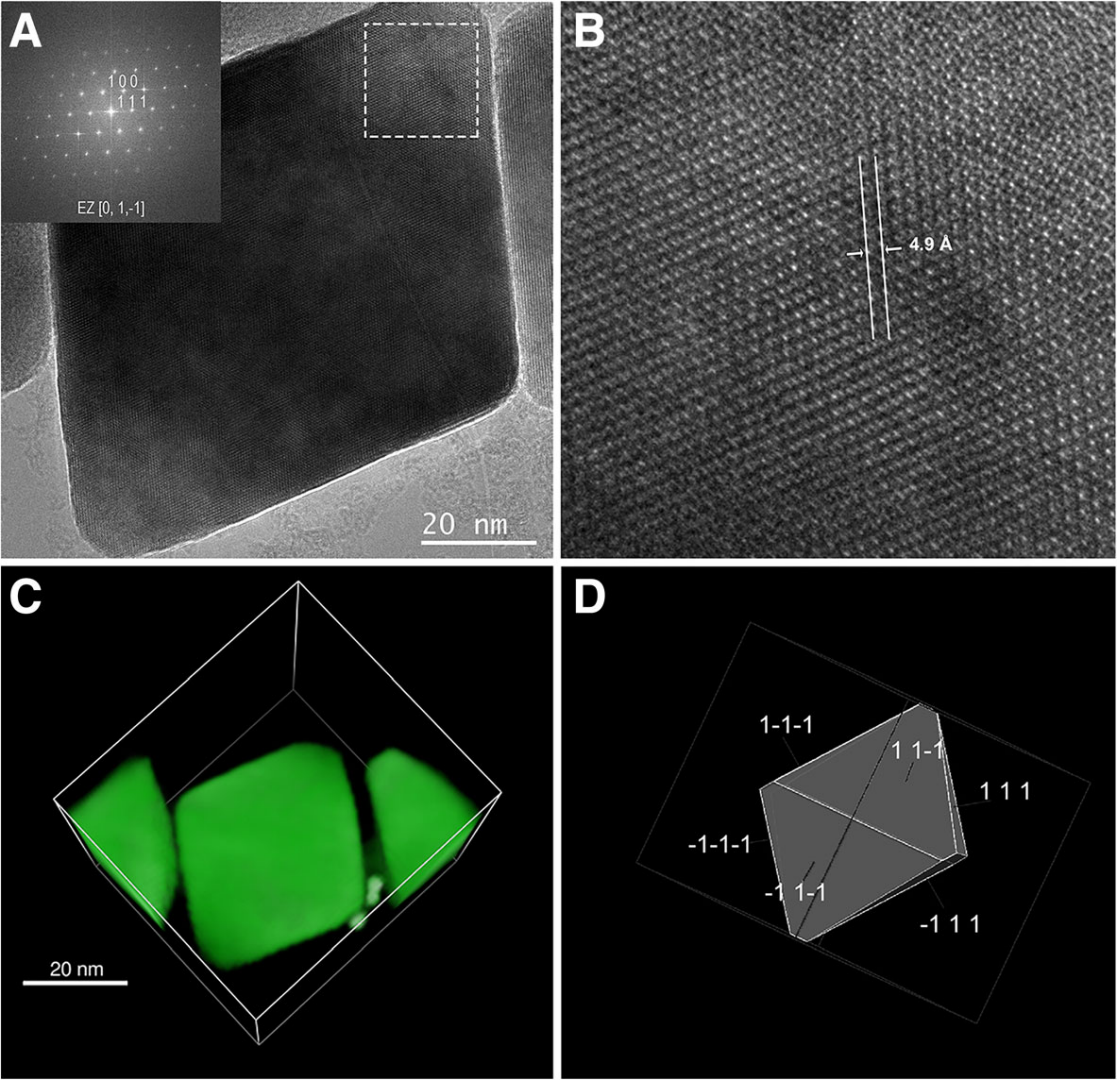
Magnetosome crystalline habit in *Mf. australis* strain IT-1. Magnetosome crystalline habit in *Mf. australis* strain IT-1. **a** High resolution transmission electron microscopy image of a single magnetosome with elongated octahedral morphology. Inset shows the Fast Fourier Transform with indexed planes and zone axis, (**b**) Higher magnification image of the dashed boxed are shown in (**a**), The spacing of fringes shown between white arrows is 4.9 Å, consistent with (1 1 1) spacing for magnetite. **c** Tomography reconstruction using STEM/HAADF of the magnetosome shown in (**a**). **d** Idealized model of magnetosome crystal in same orientation shown in (**a**)

High resolution TEM images of magnetosome crystals (Fig. 3a-b) were indexed with distances and angles between spots being consistent with cubic magnetite (Fe_3_O_4_). Tomographic analysis (Fig. 3c) was used to generate an idealized 3D model (Fig. 3d) of octahedral crystal habit elongated along [111] crystallographic direction. Magnetite crystals from magnetosome were isolated from cells growing under heterotrophic conditions with acetate as the carbon source, and averaged 90.42 ± 19.62 nm in size with an average shape factor of 0.74. These values are similar to those reported in other alphaproteobacterial MTB [38] and, although the crystal size is similar to that reported in strain MC-1, the shape factor indicates strain IT-1 has more elongated crystals, similar to those described in strains MO-1 and QH-2 [38].

*Mf. australis* strain IT-1 *mam* genes were detected in a 72,493 bp contig. Most genes from this region of the genome (a 40,399 bp fragment), particularly the *mam* genes, were previously described in Morillo et al. [13] and are more similar to homologous genes described in *Mc. marinus* strain MC-1, except *mamC*. Comparison of synteny between *Mf. australis* strain IT-1 and *Mc. marinus* strain MC-1 genomic regions containing *mam* genes is shown in Additional file 3. Besides similarities in *mam* and *mms* gene organization described by Morillo et al. [13], ORFs encoding hypothetical proteins and a protein with a PilZ domain, known for their participation in chemotaxis [39], are in relatively close proximity to the *mamAB* gene clusters, a situation similar to that of *Mc. marinus* strain MC-1. BLASTP [40] homology search shows that the only protein similar to the predicted protein containing the PilZ domain belongs to *Mc. marinus* strain MC-1 (99 % coverage, 31 % ID and 49 % positives), suggesting its possible role in magnetotaxis, by regulating speed and direction of flagellar rotation [39]. The first predicted ORFs within this contig encode two transposases (ORFs 04933, 04812) and one resolvase (ORF 04811) with no homology to *Mc. marinus* strain MC-1. At the end of the contig genes encoding two transposases (ORF 04938; 04945) and integrases (ORF 05502; 05504) were predicted; these were similar to *Mc. marinus* strain MC-1 predicted ORFs (except ORF 04945), but were not found within the putative MAI in the genome [10]. The identity between the integrases at the end of the contig (ORF 05502 and 05504) is 98 %; however, the ORF 05504 represents less than 50 % of the ORF 05502 entire sequence. This contig has 18 predicted hypothetical proteins. Most hypothetical proteins coding ORFs flanking *mam* genes [13] are only similar to *Mc. marinus* strain MC-1 predicted hypothetical proteins (identity and positive values vary from 22 to 41 % and 39 to 55 %, respectively). Although homology value is not high, the only similar sequence in the database belonged to *Mc. marinus* strain MC-1 according to BLASP analysis using NCBI non-redundant protein sequence database. The low similarity values for these predicted hypothetical proteins when compared to *Mc. marinus* strain MC-1 sequences contrast with those found for a few hypothetical proteins within the *mam* gene clusters, which identity and positive values range from 22 to 75 % and 36 to 89 %, respectively. No results were found for ORFs 02810, 04936 and 02843 based on homology search.

Magnetotaxis related genes (*mtx* genes) are localized in a contig of approximately 616 kb in size. It is not possible to predict its distance from the *mam* gene cluster, but the contig appears to be part of another cluster, as described for *Mc. marinus* strain MC-1. This cluster includes three alphaproteobacterial *mtx* genes, a Sel1 domain-containing protein coding gene, the *mtxA* gene and an adenylate/guanylate cyclase coding gene. BLASTP best hits were homologous genes described for *Mc. marinus* strain MC-1 present in an *mtx* cluster [10].

### Metabolism related genes in *Mf. australis* strain IT-1

**Fig. 4:**
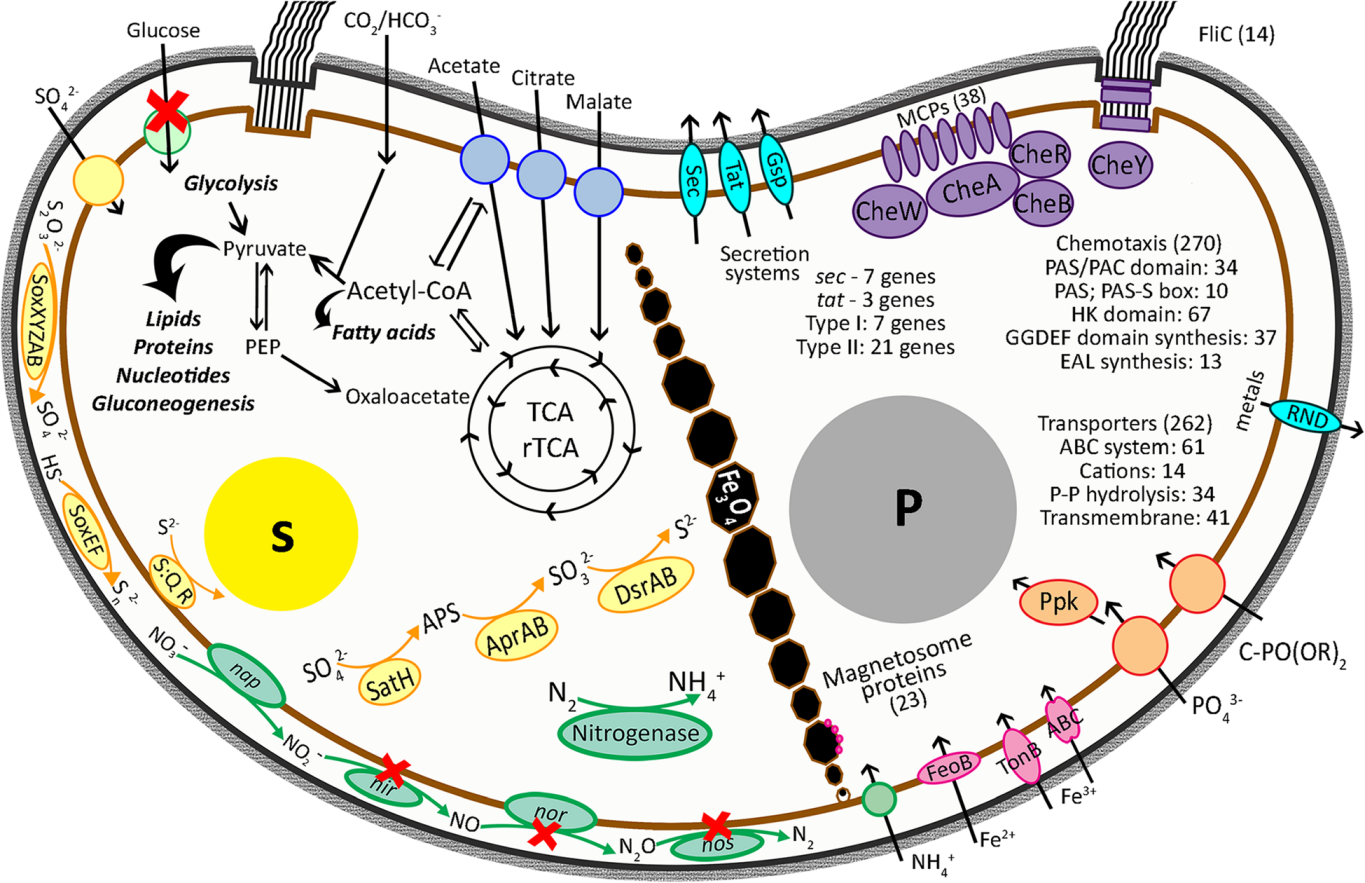
Schematic overview of *Mf. australis* strain IT-1 showing its main metabolic pathways and structural features. Schematic overview of *Mf. australis* strain IT-1 showing its main metabolic pathways and structural features. Cells are bilophotrichous with both flagella bundles in the concave face of the cell. Forty-two genes encode methyl-accepting chemoreceptors usually associated to the flagellar apparatus (*purple*). Strain IT-1 is chemolitoautotroph using the reverse tricarboxylic acids cycle (rTCA) or chemoorganoheterotroph, capable to grow using small organic molecules such as acetate and citrate. Cells are capable of nitrogen fixation, but probably do not to use nitrate as final electron acceptor (*green*). Genes for sulfate uptake and reduction were found as well as proteins responsible for sulfur compounds oxidation (*yellow*) and a gene for a sulfide:quinone reductase (S:Q R), responsible for the synthesis of sulfur globules (S). Genes encoding proteins for the synthesis of polyphosphate granules (P) are present as well as phosphate and phosphanate transporters (*orange*). High and low affinity iron transporters are encoded (*pink*) and there are copies of these genes located closely to magnetosome genes, responsible for the synthesis and organization of the chain of magnetite (Fe_3_O_4_) cubo-octahedral magnetosomes. Other cell transporters are also depicted (*blue*)

Figure 4 is an overview of *Mf. australis* strain IT-1 genes showing main the main metabolic pathways and cell components potentially used by this bacterium. Below we describe selected aspects of the genetic information related to biomineralization and metabolism.

#### Chemoreceptors, transcription and transport

Magneto-aerotaxis [5] depends on chemoreceptors capable of sensing the external or internal cell conditions. Methyl-accepting chemotaxis proteins (MCPs) are chemotactic sensors coupled to the flagellar apparatus through chemotactic enzymes CheA, CheZ, CheB and CheY, which catalyze (de-)methylation and (de-)phosphorylation reactions that result in the switching of flagella rotation either in the clockwise or anticlockwise direction [41]. According to the conserved topology of the domains and their interaction with internal membrane, MCPs have been classified into four main classes [42]. We performed analyses using SMART (Simple Modular Architecture Research Tool) [43] and the CDD (Conserved Domain Database) at NCBI [44] on locus automatically annotated as MCPs. *Mf. australis* strain IT-1 has 22 MCPs with topology of class I, three resembling a class II topology, 6 similar to class III and 7 type IV MCPs (Additional file 4).

Genomes of MTB generally contain a large number of transcription factor and transport genes presumably enabling them to modulate their magnetotactic behavior in response to diverse environmental stimuli. The large number of transduction components might be used to regulate magnetosome synthesis and magnetotactic behavior [11] and is necessary to avoid unspecific cross talk between different regulatory pathways [45].

The genome of *Mf. australis* strain IT-1 contains 272 genes encoding signal transduction proteins (Additional file 5). This number is higher than the number of transduction genes described in the genome of other magnetotactic *Alphaproteobacteria* strains. Thirty-six transduction genes in *Mf. australis* strain IT-1 are related to chemotaxis and includes four genes encoding proteins with CheW domain, three with CheA, three with CheY, two with CheB and three with CheR, two with CheB/CheR, one with CheX and 13 genes encoding hemerythrin-like metal binding proteins. These hemerythrin-like proteins are involved in oxygen transport but might have domains related to chemotaxis and signal transduction [46].

#### Iron metabolism

The first step in magnetite biomineralization is the transport of iron from the extracellular environment into the cell [47]. The genome of *Mf. australis* strain IT-1 appears to contain genes necessary for a complete and complex system for capture, transport and regulation of iron including: a large number of iron reductases; ABC-type transporters; ferritins; hemerythrins; and other proteins responsible for iron homeostasis within the cell. All of them are likely important not only for magnetosome biomineralization, but also for cell growth [48, 49].

Magnetosome genes in the genome of IT-1 are organized in a contig that also contains genes encoding hypothetical proteins, transposases, integrases, resolvases and an ORF encoding a protein with PilZ domain (ORF 02273). This contig also has ORFs coding hemerythrin-like (ORFs 02806, 02811, 05565), ferritin/ribonucleotide reductase-like (ORF 02816 and 02834), cation diffusion facilitator family transporter (ORF 02835), chromosome partitioning protein (ORF 02842), replication initiator protein A (ORF 02844) and Fis family transcriptional regulator (ORF 04937). For three ORFs encoding hemerythrins, only ORF 02811 has similarity with a gene from *Mc. marinus* strain MC-1 (35 % identity) whereas two are more similar to predicted proteins from non-MTB (ORF 02806: *Treponema brennaborense,* 35 % identity; ORF 02812: *Spirochaeta thermophile,* 34 % identity). Because hemerythrin is a protein involved in oxygen transfer, chemotaxis and signal transduction [46, 50], the hemerythrin that is common to *Mf. australis* strain IT-1 and *Mc. marinus* strain MC-1, encoded by ORF 02811, is located close to mam genes, and might be involved in magnetotaxis, as suggested [46]. Some ORFs encoding hemerythrin related proteins are also present in other contigs, all related to non-MTB sequences. Ferritin coding ORFs (ORFs 02816, 02834) are homologues to the predicted ferritin-like hypothetical protein in *Mc. marinus* strain MC-1 (34 % and 47 % identity, respectively). Ferritins are known for their function in iron detoxification, oxidation of Fe^2+^, Fe^3+^ storage and for their controlled release of iron preventing cellular toxicity [51]. If transcribed, ferritins and hemerythrins closely associated with *mam* genes could be related to magneto-aerotaxis and magnetosome synthesis [46, 51], controlling iron redox conditions on the magnetosome crystal or within the magnetosome vesicle, or the oxygen or iron flux. The presence of multiple copies of genes encoding ferritins and hemerythrins in *Mf. australis* strain IT-1 suggests their possible role in magnetosome synthesis, as previously described for *feoA* and *feoB* genes usually present in two copies in MTB genomes [52]. Two copies of the *feoAB* system were also found in *Mf. australis* strain IT-1 genome (ORFs 02827, 02828, 03818 and 03820). The best blast hits for both copies of *feoB* genes of strain IT-1 were sequences of this gene found in other magnetotactic *Alphaproteobacteria* (99 % coverage, at least 53 % identity), revealing the high similarity of this gene among MTB. One of the copies, *feoB1* (ORF 02827) is located close to mam genes and might be directly involved in magnetosome synthesis whereas *feoB2* (ORF 03818) might be involved in cell iron metabolism and detoxification of reactive oxygen species, as proposed in strain MSR-1 [49]. Two *Fur* (ferric uptake regulator) genes for Fe^3+^ uptake were also present (ORFs 04154 and 01761), one common to other alphaproteobacterial MTB and other found only in *Mc. marinus* strain MC-1. When activated by iron or other metal ions, Fur protein binds to the operator of over 90 Fur-regulated genes in *E. coli* [53]. The *Fur* gene from *Ms. gryphiswaldense* strain MSR-1 has been shown to regulate iron and oxygen metabolism [54], besides having a role in magnetosome formation [48].

The *Mf. australis* strain IT-1 genome contains genes that encode iron receptors and transporters of high and low affinity. Genes encoding CDF (cation diffusion facilitator) proteins, also found in *Mc. marinus* strain MC-1 and other magnetotactic strains are present in the genome, which also contains a gene encoding an iron receptor in outer membrane, *tonB*, and four additional genes coding TonB family proteins. Besides *mam* genes involved in redox control and iron stoichiometry (*mamE, P, T, H, Z* and *X*) during magnetosome formation, a transmembrane protein containing ferric reductase domain (ORF 03272) was found in *Mf. australis* strain IT-1. This gene has a high similarity to a putative Fe^3+^ reductase in other MTBs (*Ms. magneticum* strain AMB- 1, *Ms. gryphiswaldense* strain MSR-1, *Ms. magnetotacticum* strain MS-1, *Mc. marinus* strain MC-1). Seven genes encoding Fe-S oxidoreductases are present in *Mf. australis* strain IT-1, a large number when compared to the alphaproteobacterial magnetotactic strains MC-1, AMB-1, MSR-1 and QH-2*,* with a single gene encoding a Fe-S oxidoreductase. The genome of *Ca.* Magnetoglobus multicellularis contains 8 copies of Fe-S reductases, while strains *Ca.* Magnetoovum chiemensis and *Ca.* Magnetobacterium bavaricum have 10 and 13 copies, respectively. These cells synthesize a large number of magnetosomes and the presence of these reductases in greater number could be responsible for ensuring the availability of iron for cell growth and magnetosome synthesis.

#### Carbon metabolism

*Mf. australis* strain IT-1 grows chemoorganoheterotrophically on acetate and succinate. Chemolithoautotrophic growth occurs using sodium bicarbonate as the sole carbon source and thiosulfate as electron donor [13]. Genomic data suggest that autotrophic growth occurs via the reverse or reductive TCA cycle (rTCA) (Fig. 4), a trait shared with *Mc. marinus* strain MC-1 [10]. In other magnetotactic *Alphaproteobacteria*, including some *Magnetospirillium* species and *Mv. blakemorei* strain MV-1 [55], autotrophic growth occurs via the Calvin-Benson-Bassham cycle. In *Mf. australis* strain IT-1 genome, no predicted ORF encoding the ribulose-1,5-bisphosphate carboxylase/oxygenase (RubisCo) enzyme was detected. All enzymes required for oxidative TCA and reductive TCA cycles are encoded, but no genes encoding the enzymes necessary for the glyoxylate bypass were found.

#### Nitrogen metabolism

The genome of *Mf. australis* strain IT-1 contains all known genes necessary for nitrogen fixation, a common trait among other magnetotactic *Alphaproteobacteria*, with the exception of strain QH-2 [11]. Strain IT-1 grows in medium without addition of fixed nitrogen sources and therefore, it likely is able to fix N_2_ under favorable environmental conditions. Genes necessary for this pathway occur in two clusters, *nifZVXNEYTKDH* (ORFs 03719 to 03740, with two transposases and hypothetical proteins between *nifY* and *nifE*) and *nifQBA* (ORFs 02324, 02328 and 02330) with genes *nifR* (ORF 01176) and *nifU* (ORF 03853) occurring elsewhere in the genome. The ammonium produced by nitrogen fixation or absorbed from the environment might be assimilated through one of three pathways for which strain IT-1 carries all the necessary genes: alanine dehydrogenase (ORF 00976), glutamate dehydrogenase (ORF 03387) or glutamine synthase and glutamate synthase (ORF 03244) (GS-GOGAT cycle). These pathways are also present in the genome of *Mc. marinus* strain MC-1 [10] and in other alphaproteobacterial MTB.

Several *Magnetospirillum* species are capable of reducing certain nitrogen oxides as terminal electron acceptors for growth, producing N_2_ from nitrate through denitrification [56]. The first reaction of denitrification, the reduction of NO_3_^−^ to NO_2_^−^, is catalyzed the enzyme dissimilatory nitrate reductase, of which there are two types: a periplasmic (Nap) and a membrane-bound (Nar) form. Nap has been shown to be involved in magnetite magnetosome biomineralization, probably through redox control, in some *Magnetospirillum* species [57] and, interestingly, *nap* has been found in the genomes of all magnetotactic *Alphaproteobacteria* studied so far [7, 10, 13] including strain IT-1 (ORF 01154) as well as in the genome of the uncultured MTB *Candidatus* Magnetobacterium bavaricum from the *Nitrospirae* phylum [20]. All these MTB biomineralize magnetite and it is possible that Nap plays a role in the biomineralization of magnetite in all MTB that produce this mineral. Surprisingly, however despite possessing *nap* genes, neither *Mf. australis* strain IT-1 or *Mc. marinus* MC-1 grows anaerobically with nitrate as a terminal electron acceptor.

The genome of *Mf. australis* strain IT-1 does not contain genes for the subsequent reduction steps of denitrification (*nir*, *nor* and *nos* genes), as do the genomes of some *Magnetosprillum* species and thus it appears that it is not capable of dissimilatory nitrite, nitric oxide and nitrous oxide reduction. The situation is similar with *Mc. marinus* strain MC-1 except that there are copies of nitric oxide reductase (*norCBQ* and *norD*) in the genome of this organism [10]. Genes for the assimilatory nitrate reduction (*nas*) pathway were not found in either of the genomes of *Mf. australis* strain IT-1 or *Mc. marinus* strain MC-1 [10].

#### Sulfur metabolism

In aerobic environments, sulfur is mostly found in the oxidized form as sulfate (SO_4_^−2^) and must be reduced to be used by bacteria. The genome of *Mf. australis* strain IT-1 contains two groups of genes involved in assimilatory sulfate reduction. Two genes responsible for assimilative sulfate reduction were found, a sulfate adenylyltransferase that turns sulfate in adenylyl sulfate and other two adenylylsulfate kinases (ORFs 01705 and 03386) (Fig. 4). *Mf. australis* strain IT-1 does not have genes that encode the proteins CysH and CysI, capable of catalyzing 3′-phospho-5′-adenylyl sulfate to sulfite and reducing sulfite to H_2_S, respectively.

ORFs encoding enzymes involved in dissimilatory sulfate reduction sulfate to sulfite (dissimilatory sulfate reductase – DsrAB and adenylyl-sulfate reductase – AprAB) are present in the genome of *Mf. australis* strain IT-1. We found six *dsr* genes in the *Mf. australis* strain IT-1 genome, *dsrAB* (ORFs 03242 and 03243)*, dsrC* (ORF 00945) and *dsrHFE* (ORFs 03513 to 03515) that are clustered close to two sulfite oxidase genes, *yedY* and *yedZ* (ORFs 03271 and 03272, respectively). This arrangement of the *dsr* and *yed* genes is also similar in the genomes of *Ms. magneticum* strain AMB-1 and *Mc. marinus* strain MC-1. In some bacteria, such as *Allochromatium vinosum*, the Dsr (dissimilatory sulfite reductase) proteins are essential for the oxidation of zero-valent sulfur in sulfur globules [58]. We have also found a gene encoding a sulfate adenylyltransferase (ORF 02663) with higher similarity to a marine *Gammaproteobacteria* (accession number: WP007226305, 99 % coverage, 78 % ID and 89 % positives) and genes encoding adenylyl-sulfate reductase subunits (AprAB, ORFs 2173 and 3566, respectively) with higher similarity to *Thiocystis violascens* (accession number: WP_014779579, 99 % coverage, 67 % ID and 77 % positives) and to *Acromatium* sp. (accession number: KOR32136, 98 % coverage, 85 % ID and 94 % positives), respectively. However, we could not unequivocally detect genes encoding the QmoABC membrane complex, responsible for electron transfer to the AprAB enzyme in sulfate reducing bacteria [59]. Under laboratory conditions, *Mf. australis* strain IT-1 is capable of oxidizing sulfide but does not grow anaerobically with sulfate as the sole terminal electron acceptor, however this feature would confer an important ecological advantage for *Mf. australis* strain IT-1, since the Itaipu lagoon receives high organic matter loads that decrease O_2_ availability and as it is a marine habitat, the water is rich in sulfate.

The genome of *Mf. australis* strain IT-1 contains two set of *sox* genes. Enzymes for this pathway are responsible for the oxidation of reduced sulfur compounds directly to sulfate, without a sulfite intermediate. The first set of genes is comprised of *soxXYZAB* (ORFs 03354 to 03358) and is organized similarly in the genome of *Mc. marinus* strain MC-1. The second set consists of *soxEFXY* (ORFs 02177 to 02182). The *soxW* (ORF 01228) gene is distant from the other *sox* genes. Two copies of *soxY*, that encodes an enzyme that binds the sulfur compound to be oxidized, are present (ORFs 02181 and 03355). However, *soxC* and *soxD*, both encoding for proteins involved in electron transfer chain, are not present in *Mf. australis* strain IT-1. Genes encoding a sulfide quinone reductase (ORFs 01515, 02178 and 02650), a key enzyme for sulfur globule synthesis, are present in *Mf. australis* strain IT-1 and confirms its ability to produce intracellular sulfur globules (Fig. 4), as observed by microscopy (see Fig. 1; [13]). Other genes encoding proteins that directly or indirectly act in sulfur metabolism are found dispersed in *Mf. australis* strain IT-1 genome and include three specific sulfate transporter (ORFs 00291, 02332 and 02706), one bifunctional sulfur transporter/tiasol synthase, four sulfotransferase and seven ABC transporters for nitrate/sulfonate/bicarbonate.

#### Phosphorous metabolism

Phosphorus, generally in the form of phosphate, plays a major role in diverse cellular processes and efficient control over the uptake and storage of phosphate and the regulation of phosphate metabolism is mandatory. The genome of *Mf. australis* strain IT-1 contains genes related to the *Pho* regulon (two copies of *phoU* and *phoBR*, ORFs 0718, 0148; 03223 and 03224), responsible for the uptake of phosphate, as well as genes (ORFs 01828 and 03685) regulating the absorption of phosphonates [60, 61]. Genes encoding the high affinity transport of phosphorus (*pstBAC*, ORF 04289, 04291 and 04292; *pstS1* and *pstS2,* ORF 02354 and 03453), a permease (ORF 04293), three phosphonate transporters and a diguanylate cyclase/phosphodiesterase (ORF 04288) also occur in the genome of IT-1. A similar arrangement is present in the genome of *Mc. marinus* strain MC-1. This type of regulation has also been described for *Magnetospirillum* strains AMB-1, MS-1 and MSR-1 [10]. Both MC-1 and IT-1 have genes encoding polyphosphate kinase and exopolyphosphatase enzymes, both involved in the synthesis of polyphosphate granules [62] (Fig. 4). Polyphosphate granules function as an energy and phosphate reservoir as well as being involved in kinase reactions [63]. It has been shown that a lack of polyphosphate kinase hampers the cell response to environmental stress [64–66].

Phosphate metabolism may also be related to magnetosome biosynthesis. Phosphate granules are widespread in magnetotactic cocci whether sampled from the environment [67] or grown in culture even when the phosphate concentration is relatively low (Fig. 1, [1, 10]). It has been shown the involvement of a phosphate-rich ferric hydroxide phase for storage of iron inside magnetosome vesicle before magnetite crystals formation [68].

#### Detoxification of reactive oxygen species

The genome of *Mf. australis* strain IT-1 contains 9 ORFs encoding proteins involved in detoxifying reactive oxygen species. There are four ORFs encoding cytochrome C peroxidase (ORFs 00488, 02063, 03177 and 03947), three encoding alkyl hydroperoxide reductase (ORFs 02341, 02949 and 03103), one catalase (ORF 01369) and one superoxide dismutase (02343). This number of ORFs is lower than that encoded in the genome of *Ms. magneticum* strain AMB-1 (15) but much higher than the 3 genes encountered in the *Mc. marinus* strain MC-1 genome, implying that *Mf. australis* might be more resistant to oxidative stress than the marine magnetotactic cocci.

## Conclusions

The characterization of MTB, whether cultured or uncultured, is largely based on morphological, behavioral, metabolic and genomic aspects. Here we used phenotypic and genotypic data to describe features in *Mf. australis* strain IT-1, a cultured magnetotactic coccus, associated with its metabolism, its ability to produce magnetosomes, and its structure and behavior. The unique alignment of the linear magnetosome chain with flagella bundles may indicate the rotating cell body observed associated with a high speed swimming may help *Mf. australis* strain IT-1 to overcome physical barriers of objects encountered in sediment or help to quickly move away detrimental stimuli. Environmental conditions in Itaipu lagoon change constantly and the high number of genes encoding transport and transduction genes in strain IT-1 favors its survival. Genomic analysis indicates *Mf. australis* strain IT-1 and *Mc. marinus* strain MC-1 are closely related species but contain some different, distinctive features. Similarity values between the amino acid sequences of homologous proteins are not very high and genomic sequencing of other magnetotactic cocci such the marine bacterium strain MO-1 will improve our knowledge regarding the magnetotactic cocci in general based on genomic analyses.

## Methods

*Mf. australis* strain IT-1 was routinely cultured in a semisolid, heterotrophic medium [13]. High-speed imaging was done on a Nikon Eclipse Ti microscope equipped with a CFI Plan Apo 100x/1.45 objective lens and attached to a Photron Model 675K-M1 Fastcam camera (Photron, San Diego, CA). Images were analyzed using the free ImageJ software (rsb.info.nih.gov/ij/). Scanning electron microscopy was performed as described in [69]. For high pressure freezing and freeze substitution, a culture of *Mf. australis* strain IT-1 was diluted in artificial seawater and magnetically concentrated. For freeze substitution, samples were quick-frozen with a Leica HPM 100 high pressure freezing apparatus (Leica Microsystems, Bannockburn, IL, USA) and transferred to a fixative solution containing 2 % osmium tetroxide and 0.1 % uranyl acetate in anhydrous acetone. Samples were kept at -90 °C for 90h, then at -35 °C for 4 h and -20 °C for 2h. The temperature was gradually increased to room temperature and samples were embedded and polymerized in PolyBed 812. Ultrathin sections were obtained on Leica EM U6 ultramicrotome (Leica Microsystems, Bannockburn, IL, USA), stained with uranyl acetate and lead citrate and imaged with a Morgagni transmission electron microscope (FEI Company, Hillsboro, OR, USA) at 80kV. Electron tomography was performed using a JEOL JEM 2100F transmission electron microscope operated at 200 kV using STEM/HAADF mode. Tomographic tilt series were acquired by tilting the samples from at least -60° to +60° at 1.46° intervals. Fast Fourier Transform (FFT) was obtained from high-resolution calibrated images using Digital Micrograph software (Gatan Inc.). 3D models of the crystals were constructed using KrystalShaper program (JCrystalSoft). Electron tomography was aligned and reconstructed using software ImageJ (http://rsb.info.nih.gov/ij/) with the plug-in TomoJ. 3D model was obtained using UCSF Chimera software (University of California, San Francisco, http://www.cgl.ucsf.edu/chimera/).

For DNA preparation and pyrosequencing, cells were concentrated by centrifugation and washed several times with sterile distilled water. DNA was sequenced on a 454 GS FLX System sequencer (Roche Diagnostics GmbH/454 Life Sciences Corporation, Branford, CT, USA) and the assembly was performed using WGS-Assembler v7.0 and Newbler v2.8, and the contigs were joined with homemade scripts. The genome has been analyzed with SABIA (System for Automated Bacterial Integrated Annotation) platform [70]. Gene prediction was obtained with the Glimmer and genes were annotated using blast (query and subject coverage equal to 60 % and positives equal 50 %) and KEGG database. Mam genes synteny were analyzed based on the Bidirectional Best Hit (BBH) approach using BLASTP Program. All BLASTP searches were performed with an e-value equal 1e-05, query coverage equal 60 % and similarity equal 50 % [40].

## Additional files

Additional file 1: Magnetotactic bacteria genomes (March 2016) [6–22]. (DOCX 15 kb)
Additional file 2: General information on *Mf. australis* strain IT-1 genome sequence. (DOCX 12 kb)
Additional file 3: Comparison of *Mf. australis* contig containing mam genes and the putative MAI of *Mc. marinus*. Schematic comparison of *Mf. australis* strain IT-1 contig containing mam genes and its corresponding region in the putative MAI of *Mc. marinus* strain MC-1. Similar regions are marked in boxes (pink, yellow, blue and gray). Grey ORFs represent those that encode non-hypothetical proteins. Annotation was omitted at the end of the putative MAI of *Mc. marinus* strain MC-1 because it does not correspond to any region of *Mf. australis* strain IT-1 present above. tnp: transposase; rsl: resolvase; HP: hypothetical protein; aa carrier: amino acid carrier protein; mcp: methyl-accepting chemotaxis protein; hm: hemerythrin-like protein; ft: ferritin-like protein; fis: Fis family transcriptional regulator; int: integrase; par: chromosome partitioning protein; trfA: replication initiator protein A. Unique *Mf. australis* strain IT1 hypothetical proteins are labeled with asterisks. (PNG 365 kb)
Additional file 4: Classes of MCPs annotated in *Mf. australis*. Conserved sensory or ligand binding regions are presented. (DOCX 12 kb)
Additional file 5: Genes involved in signal transduction in *Mf. australis* strain IT-1. (DOCX 25 kb)

## Abbreviations

ABC transporter: ATP-binding cassette transporters
BLASTP: Basic local alignment search tool protein
bp: Base pair
CCD Camera: Charge-coupled device camera
CDD: Conserved domain database
CDF: Cation diffusion facilitator
FFT: Fast fourier transform
G + C: Guanidine and cytosine content
GS-GOGAT cycle: Glutamine synthetase-glutamine oxoglutarate aminotransferase cycle
HAADF: High angle annular dark field
KEGG: Kyoto encyclopedia of genes and genomes
*mam* genes: Magnetosome membrane genes
MCP: Methyl-accepting chemotaxis proteins
mms genes: Magnetosome membrane-specific genes
MTB: Magnetotactic bacteria
mtx genes: Magnetotaxis related genes
NCBI: National center for biotechnology information
ORF: Open reading frame
SMART: Simple modular architecture research tool
STEM: Scanning transmission electron microscope
TCA/rTCA: Tricarboxylic acids cycle/ reverse tricarboxylic acids cycle
WGS: Whole genome shotgun
