# Erratum to: Combined genomic and structural analyses of a cultured magnetotactic bacterium reveals its niche adaptation to a dynamic environment

**DOI:** 10.1186/s12864-016-3243-8

**Published:** 2016-11-09

**Authors:** Ana Carolina Vieira Araujo, Viviana Morillo, Jefferson Cypriano, Lia Cardoso Rocha Saraiva Teixeira, Pedro Leão, Sidcley Lyra, Luiz Gonzaga de Almeida, Dennis A. Bazylinski, Ana Tereza Ribeiro de Vasconcelos, Fernanda Abreu, Ulysses Lins

**Affiliations:** 1 Instituto de Microbiologia Paulo de Góes, Universidade Federal do Rio de Janeiro, 21941-902 Rio de Janeiro, RJ Brazil; 2 School of Life Sciences, University of Nevada at Las Vegas, Las Vegas, NV 89154-4004 USA; 3 Departamento de Matemática Aplicada e Computacional, Laboratório Nacional de Computação Científica, 25651-070 Petrópolis, RJ Brazil; 4 Current institution: Departamento de Biologia, Universidade Federal de São Carlos, 18052-780 Sorocaba, SP Brazil

## Erratum

Following publication of the original article [1] it was brought to our attention that one of the authors’ names had been incorrectly spelled. The ninth author name, Ana Tereza Ribeiro de Vasconcelos, was originally written incorrectly as Ana Tereza Ribeiro de Vasconcellos. Please note that the correct spelling should be Ana Tereza Ribeiro de Vasconcelos.
