# Supplementary material for: Combined genomic and structural analyses of a cultured magnetotactic bacterium reveals its niche adaptation to a dynamic environment
Source: BMC Genomics. 2016 Oct 25;17(Suppl 8):726. doi: 10.1186/s12864-016-3064-9 (PMC5088516; doi:10.1186/s12864-016-3064-9)
Supplement: Additional file 1: — Magnetotactic bacteria genomes (March 2016) [6–22]. (DOCX 15 kb) [file 12864_2016_3064_MOESM1_ESM.docx]

| **Additional file 1.** Magnetotactic bacteria genomes (March 2016) | | | | | | | |
| --- | --- | --- | --- | --- | --- | --- | --- |
|  | **Bacteria** | **Accession number** | **Status** | **Genome size (bp)** | **Plasmid** | **GC content** | **Reference** |
| **Alphaproteobacteria** | *Magnetospirillum gryphiswaldense* strain MSR-1 | HG794546 | Complete | 4,365,796 | - | 63.28 | [6] |
|  | *Magnetospirillum magneticum*  strain AMB-1 | AP007255 | Complete | 4,967,148 | - | 65.09 | [7] |
|  | *Magnetospirillum magnetotacticum* strain MS-1 | JXSL00000000 | Partial  36 contigs | 4,523,935 | - | 63.6 | [8] |
|  | *Magnetospirillum caucaseum*  strain SO-1 | AONQ01000000 | Partial  261 contigs | 4,874,064 | - | 65.98 | [9] |
|  | *Magnetospirillum sp.*  strain XM-1 | PRJEB11958 | Complete | 4,825,187 | 1 (167,290 bp) | 65.6 (chr)  66.5 (plsm) | unpublished |
|  | *Magnetococcus marinu*s strain MC-1 | CP000471 | Complete | 4,719,581 | - | 54.17 | [10] |
|  | *Magnetofaba australis* strain IT-1 | KF933436 | Partial  *mam* genes contig | 72,493 | - | 57.6 | [13] |
|  | *Magnetofaba australis* strain IT-1 | LVJN01000000 | Partial  21 contigs | 4,986,701 | - | 57.95 | This work |
|  | *Magnetovibrio blakemorei*  strain MV-1 | FP102531 | Partial  *mam* genes contig | 107,223 | - | 55.8 | [14] |
|  | *Magnetospira sp.*  strain QH-2 | PRJEB1386 | Complete | 4,020,900 | 1 (31,063 bp) | 59.5 (chr)  54.8 (plsm) | [11] |
| **Deltaproteobacteria** | *Desulfovibrio magneticus*  strain RS-1 | Genome NCBI ID 1358 | Complete | 5,248,049 | 1 (58,704 bp)  2 (8,867 bp) | 62.8 (chr)  58.0 (plsm1)  37.2 (plsm2) | [12] |
|  | *Ca.* Magnetoglobus multicellularis* | PRJNA52963 | Partial  3,706 contigs | 12,453,800 | - | 37.3 | [15] |
|  | *Ca. Magnetomorum*  strain HK-1 | PRJNA252699 | Partial  3,036 contigs | 14,290,400 | - | 34.7 | [16] |
|  | *Ca.* Desulfamplus magnetomortis strain BW-1 | PRJEB589 | Partial  *mam* genes | 254,780 | - | 41.0 | [17] |
|  | Deltaproteobacterium strain ML-1 | JX869936  JX869937 | Partial  (*mam* genes) | 13,272  21,411 | - | 61.7  62.3 | [18] |
| **Nitrospirae** | *Ca.* Magnetobacterium bavaricum** | LACI00000000 | Partial  2,752 contigs | 6,310,440 | - | 47.3 | [19] |
|  | *Ca.* Magnetoovum chiemensis** | JZJI00000000 | Partial  1,019 contigs | 3,816,930 | - | 40.4 | [19] |
|  | *Ca.* Magnetobacterium casensis** | JMFO00000000 | Partial  70 contigs | 3,415,680 | - | 48.9 | [20] |
| **PVC superphylum** | *Ca*. Omnitrophus magneticus SKK-01** | JYNY00000000 | Partial  656 contigs | 3,145,900 | - | 35.8 | [19] |
| **Latescibacteria** | *Latescibacteria bacterium*  SCGC AAA252-B13** | ASWY00000000 | Partial  138 contigs | 1,759,980 | - | 40.9 | [21]  [22] |

* Magnetically enriched cells from environmental samples

** Single cell genomics
