# Supplementary material for: Combined genomic and structural analyses of a cultured magnetotactic bacterium reveals its niche adaptation to a dynamic environment
Source: BMC Genomics. 2016 Oct 25;17(Suppl 8):726. doi: 10.1186/s12864-016-3064-9 (PMC5088516; doi:10.1186/s12864-016-3064-9)
Supplement: Additional file 2: — General information on Mf. australis strain IT-1 genome sequence. (DOCX 12 kb) [file 12864_2016_3064_MOESM2_ESM.docx]

**Additional file 2.** General information on *Mf. australis* strain IT-1 genome sequence.

| Genome coverage | 33 X |
| --- | --- |
| Length (bp) | 4,986,701 |
| G+C content (%) | 57.95 |
| Coding density (%) | 82.64 |
| Average of ORF length (bp) | 1,010 |
| Total number of loci | 4,130 |
| Number of known protein ORFs | 2,886 |
| Number of hypothetical ORFs | 1,194 |
| Rrna |  |
| rRNA 16s | 2 |
| rRNA 23s | 2 |
| rRNA 5s | 2 |
| tRNA | 44 |
| KEGG matches | 3,693 |
| InterPro matches | 3,288 |
