# Supplementary figures and images for: Combined genomic and structural analyses of a cultured magnetotactic bacterium reveals its niche adaptation to a dynamic environment
Source: BMC Genomics. 2016 Oct 25;17(Suppl 8):726. doi: 10.1186/s12864-016-3064-9 (PMC5088516; doi:10.1186/s12864-016-3064-9)

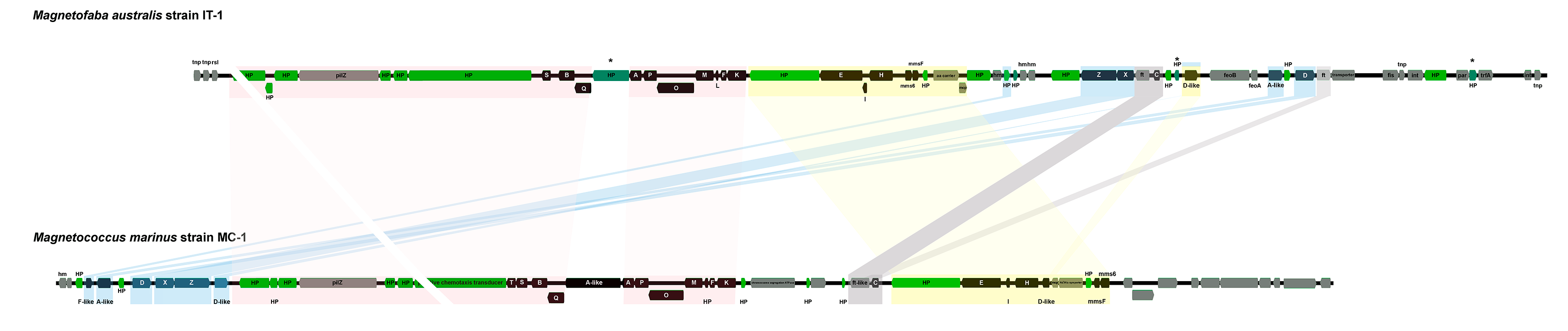

Supplement: Additional file 3: — Comparison of Mf. australis contig containing mam genes and the putative MAI of Mc. marinus. Schematic comparison of Mf. australis strain IT-1 contig containing mam genes and its corresponding region in the putative MAI of Mc. marinus strain MC-1. Similar regions are marked in boxes (pink, yellow, blue and gray). Grey ORFs represent those that encode non-hypothetical proteins. Annotation was omitted at the end of the putative MAI of Mc. marinus strain MC-1 because it does not correspond to any region of Mf. australis strain IT-1 present above. tnp: transposase; rsl: resolvase; HP: hypothetical protein; aa carrier: amino acid carrier protein; mcp: methyl-accepting chemotaxis protein; hm: hemerythrin-like protein; ft: ferritin-like protein; fis: Fis family transcriptional regulator; int: integrase; par: chromosome partitioning protein; trfA: replication initiator protein A. Unique Mf. australis strain IT1 hypothetical proteins are labeled with asterisks. (PNG 365 kb) [file 12864_2016_3064_MOESM3_ESM.png]
