# Supplementary material for: Combined genomic and structural analyses of a cultured magnetotactic bacterium reveals its niche adaptation to a dynamic environment
Source: BMC Genomics. 2016 Oct 25;17(Suppl 8):726. doi: 10.1186/s12864-016-3064-9 (PMC5088516; doi:10.1186/s12864-016-3064-9)
Supplement: Additional file 4: — Classes of MCPs annotated in Mf. australis. Conserved sensory or ligand binding regions are presented. (DOCX 12 kb) [file 12864_2016_3064_MOESM4_ESM.docx]

**Additional file 4.** Classes of MCPs annotated in *Mf. australis* strain IT-1 genome. Conserved sensory or ligand binding regions are presented.

|  | **Sensory domain** | **4HB_MCP** | **Cache** | **DUF3365** | **Globin-like** | **HBM** | **NIT** | **Tar-Tsr-LDB** | **Unknown** | **Total** |
| --- | --- | --- | --- | --- | --- | --- | --- | --- | --- | --- |
| **MCP Type** | **I** | 3 | 3 | 1 | 0 | 2 | 1 | 1 | 11 | 22 |
|  | **II** | 0 | 1 | 0 | 0 | 1 | 0 | 0 | 1 | 3 |
|  | **III** | 0 | 0 | 0 | 0 | 0 | 0 | 1 | 5 | 6 |
|  | **IV** | 0 | 0 | 2 | 1 | 0 | 0 | 0 | 4 | 7 |
|  | **Total** | 3 | 4 | 3 | 1 | 3 | 1 | 2 | 21 | **38** |
