# Supplementary material for: Combined genomic and structural analyses of a cultured magnetotactic bacterium reveals its niche adaptation to a dynamic environment
Source: BMC Genomics. 2016 Oct 25;17(Suppl 8):726. doi: 10.1186/s12864-016-3064-9 (PMC5088516; doi:10.1186/s12864-016-3064-9)
Supplement: Additional file 5: — Genes involved in signal transduction in Mf. australis strain IT-1. (DOCX 25 kb) [file 12864_2016_3064_MOESM5_ESM.docx]

**Additional file 5.** Genes involved in signal transduction in *Mf. australis* strain IT-1.

| **Locus** | **Protein** | **Length (aa)** | |
| --- | --- | --- | --- |
| 131 | adenylate cyclase | | 433 |
| 2093 | adenylate cyclase | | 642 |
| 44 | adenylate/guanylate cyclase | | 296 |
| 80 | adenylate/guanylate cyclase | | 712 |
| 935 | adenylate/guanylate cyclase | | 696 |
| 1184 | adenylate/guanylate cyclase | | 694 |
| 1708 | adenylate/guanylate cyclase | | 549 |
| 2024 | adenylate/guanylate cyclase | | 685 |
| 2240 | adenylate/guanylate cyclase | | 329 |
| 2682 | adenylate/guanylate cyclase | | 999 |
| 3219 | adenylate/guanylate cyclase | | 601 |
| 3399 | adenylate/guanylate cyclase | | 734 |
| 4247 | adenylate/guanylate cyclase | | 734 |
| 617 | Aerobic respiration control sensor protein ArcB | | 1001 |
| 95 | Autoinducer 2 sensor kinase/phosphatase luxQ | | 796 |
| 4757 | Autoinducer 2 sensor kinase/phosphatase luxQ | | 883 |
| 3292 | CheA signal transduction histidine kinase | | 937 |
| 4089 | chemotaxis CheB/CheR fusion protein | | 1095 |
| 678 | chemotaxis protein CheA | | 914 |
| 1273 | chemotaxis protein CheA | | 814 |
| 679 | chemotaxis protein CheB | | 353 |
| 675 | chemotaxis protein CheR | | 495 |
| 1268 | chemotaxis protein CheR | | 334 |
| 3296 | Chemotaxis response regulator protein-glutamate methylesterase 2 | | 583 |
| 674 | CheW protein | | 108 |
| 676 | CheW protein | | 199 |
| 1272 | CheW protein | | 170 |
| 3294 | CheW protein | | 359 |
| 3825 | CheW protein | | 164 |
| 224 | CheY-like chemotaxis protein, response regulator receiver | | 139 |
| 1934 | cyclic nucleotide-binding protein | | 318 |
| 2261 | cyclic nucleotide-binding protein | | 322 |
| 79 | diguanylate cyclase | | 622 |
| 267 | diguanylate cyclase | | 481 |
| 344 | diguanylate cyclase | | 682 |
| 370 | diguanylate cyclase | | 451 |
| 535 | diguanylate cyclase | | 554 |
| 1109 | diguanylate cyclase | | 923 |
| 1307 | diguanylate cyclase | | 491 |
| 1355 | diguanylate cyclase | | 495 |
| 1539 | diguanylate cyclase | | 446 |
| 2674 | diguanylate cyclase | | 320 |
| 2716 | diguanylate cyclase | | 561 |
| 2965 | diguanylate cyclase | | 452 |
| 2987 | diguanylate cyclase | | 462 |
| 3135 | diguanylate cyclase | | 422 |
| 3180 | diguanylate cyclase | | 687 |
| 3463 | diguanylate cyclase | | 336 |
| 4640 | diguanylate cyclase | | 421 |
| 4645 | diguanylate cyclase | | 359 |
| 2089 | diguanylate cyclase/phosphodiesterase | | 772 |
| 2385 | diguanylate cyclase/phosphodiesterase | | 1248 |
| 4288 | diguanylate cyclase/phosphodiesterase | | 563 |
| 4644 | diguanylate cyclase/phosphodiesterase with PAS/PAC sensor | | 753 |
| 555 | DNA-binding response regulator | | 221 |
| 251 | GGDEF domain protein | | 389 |
| 2463 | GGDEF domain-containing protein | | 391 |
| 353 | histidine kinase | | 807 |
| 413 | histidine kinase | | 620 |
| 558 | histidine kinase | | 430 |
| 1824 | histidine kinase | | 754 |
| 2082 | histidine kinase | | 648 |
| 2331 | histidine kinase | | 994 |
| 3184 | histidine kinase | | 899 |
| 3784 | histidine kinase | | 630 |
| 4356 | histidine kinase | | 731 |
| 1773 | Histidine phosphotransfer protein isoform 1 | | 121 |
| 3282 | Hpt protein | | 101 |
| 589 | hypothetical protein | | 545 |
| 680 | hypothetical protein | | 141 |
| 700 | hypothetical protein | | 145 |
| 704 | hypothetical protein | | 124 |
| 1269 | hypothetical protein | | 172 |
| 1288 | hypothetical protein | | 137 |
| 2234 | hypothetical protein | | 700 |
| 2353 | hypothetical protein | | 663 |
| 2389 | hypothetical protein | | 328 |
| 3020 | hypothetical protein | | 183 |
| 3283 | hypothetical protein | | 196 |
| 3623 | hypothetical protein | | 1922 |
| 3652 | hypothetical protein | | 699 |
| 4718 | hypothetical protein | | 762 |
| 89 | integral membrane sensor hybrid histidine kinase | | 661 |
| 3353 | LuxR family transcriptional regulator | | 214 |
| 4086 | LuxR family transcriptional regulator | | 203 |
| 3599 | metal dependent phosphohydrolase | | 481 |
| 685 | methyl-accepting chemotaxis protein | | 1261 |
| 91 | methyl-accepting chemotaxis sensory transducer | | 985 |
| 275 | methyl-accepting chemotaxis sensory transducer | | 716 |
| 452 | methyl-accepting chemotaxis sensory transducer | | 672 |
| 480 | methyl-accepting chemotaxis sensory transducer | | 677 |
| 572 | methyl-accepting chemotaxis sensory transducer | | 931 |
| 677 | methyl-accepting chemotaxis sensory transducer | | 524 |
| 698 | methyl-accepting chemotaxis sensory transducer | | 1081 |
| 1225 | methyl-accepting chemotaxis sensory transducer | | 613 |
| 1254 | methyl-accepting chemotaxis sensory transducer | | 887 |
| 1348 | methyl-accepting chemotaxis sensory transducer | | 666 |
| 1367 | methyl-accepting chemotaxis sensory transducer | | 700 |
| 1746 | methyl-accepting chemotaxis sensory transducer | | 540 |
| 1747 | methyl-accepting chemotaxis sensory transducer | | 482 |
| 1750 | methyl-accepting chemotaxis sensory transducer | | 812 |
| 1854 | methyl-accepting chemotaxis sensory transducer | | 785 |
| 1884 | methyl-accepting chemotaxis sensory transducer | | 777 |
| 2053 | methyl-accepting chemotaxis sensory transducer | | 905 |
| 2085 | methyl-accepting chemotaxis sensory transducer | | 805 |
| 2219 | methyl-accepting chemotaxis sensory transducer | | 677 |
| 2356 | methyl-accepting chemotaxis sensory transducer | | 757 |
| 2620 | methyl-accepting chemotaxis sensory transducer | | 610 |
| 2686 | methyl-accepting chemotaxis sensory transducer | | 784 |
| 3070 | methyl-accepting chemotaxis sensory transducer | | 755 |
| 3293 | methyl-accepting chemotaxis sensory transducer | | 480 |
| 3345 | methyl-accepting chemotaxis sensory transducer | | 932 |
| 3437 | methyl-accepting chemotaxis sensory transducer | | 210 |
| 3518 | methyl-accepting chemotaxis sensory transducer | | 726 |
| 3559 | methyl-accepting chemotaxis sensory transducer | | 862 |
| 3743 | methyl-accepting chemotaxis sensory transducer | | 822 |
| 3797 | methyl-accepting chemotaxis sensory transducer | | 856 |
| 3864 | methyl-accepting chemotaxis sensory transducer | | 840 |
| 3883 | methyl-accepting chemotaxis sensory transducer | | 762 |
| 4074 | methyl-accepting chemotaxis sensory transducer | | 831 |
| 4611 | methyl-accepting chemotaxis sensory transducer | | 765 |
| 4646 | methyl-accepting chemotaxis sensory transducer | | 661 |
| 584 | multi-sensor hybrid histidine kinase | | 744 |
| 656 | multi-sensor hybrid histidine kinase | | 1075 |
| 753 | multi-sensor hybrid histidine kinase | | 2956 |
| 905 | multi-sensor hybrid histidine kinase | | 990 |
| 1224 | multi-sensor hybrid histidine kinase | | 745 |
| 1336 | multi-sensor hybrid histidine kinase | | 935 |
| 1337 | multi-sensor hybrid histidine kinase | | 1465 |
| 1552 | multi-sensor hybrid histidine kinase | | 593 |
| 1864 | multi-sensor hybrid histidine kinase | | 762 |
| 2064 | multi-sensor hybrid histidine kinase | | 1284 |
| 2108 | multi-sensor hybrid histidine kinase | | 917 |
| 2308 | multi-sensor hybrid histidine kinase | | 931 |
| 3112 | multi-sensor hybrid histidine kinase | | 1150 |
| 3220 | multi-sensor hybrid histidine kinase | | 677 |
| 3707 | multi-sensor hybrid histidine kinase | | 1152 |
| 3839 | multi-sensor hybrid histidine kinase | | 1202 |
| 3959 | multi-sensor hybrid histidine kinase | | 677 |
| 4233 | multi-sensor hybrid histidine kinase | | 650 |
| 2521 | multisensor signal transduction histidine kinase | | 223 |
| 3224 | multi-sensor signal transduction histidine kinase | | 587 |
| 1178 | nitrogen metabolism transcriptional regulator, NtrC, Fis family | | 498 |
| 565 | PAS domain S-box | | 1164 |
| 1948 | PAS domain S-box | | 671 |
| 3349 | PAS domain S-box | | 1100 |
| 4091 | PAS domain S-box | | 919 |
| 681 | PAS domain-containing protein | | 1564 |
| 1237 | PAS domain-containing protein | | 995 |
| 2555 | PAS domain-containing protein | | 815 |
| 3097 | PAS domain-containing protein | | 1039 |
| 3693 | PAS domain-containing protein | | 986 |
| 3733 | PAS domain-containing protein | | 1515 |
| 211 | PAS/PAC sensor hybrid histidine kinase | | 741 |
| 305 | PAS/PAC sensor hybrid histidine kinase | | 1482 |
| 346 | PAS/PAC sensor hybrid histidine kinase | | 813 |
| 416 | PAS/PAC sensor hybrid histidine kinase | | 1020 |
| 487 | PAS/PAC sensor hybrid histidine kinase | | 833 |
| 690 | PAS/PAC sensor hybrid histidine kinase | | 1098 |
| 702 | PAS/PAC sensor hybrid histidine kinase | | 1071 |
| 1653 | PAS/PAC sensor hybrid histidine kinase | | 800 |
| 2220 | PAS/PAC sensor hybrid histidine kinase | | 956 |
| 2305 | PAS/PAC sensor hybrid histidine kinase | | 1925 |
| 2524 | PAS/PAC sensor hybrid histidine kinase | | 1400 |
| 3129 | PAS/PAC sensor hybrid histidine kinase | | 800 |
| 3352 | PAS/PAC sensor hybrid histidine kinase | | 711 |
| 3892 | PAS/PAC sensor hybrid histidine kinase | | 924 |
| 3986 | PAS/PAC sensor hybrid histidine kinase | | 1366 |
| 4090 | PAS/PAC sensor hybrid histidine kinase | | 562 |
| 4155 | PAS/PAC sensor hybrid histidine kinase | | 903 |
| 4326 | PAS/PAC sensor hybrid histidine kinase | | 874 |
| 37 | PAS/PAC sensor signal transduction histidine kinase | | 766 |
| 588 | PAS/PAC sensor signal transduction histidine kinase | | 704 |
| 4472 | PAS/PAC sensor signal transduction histidine kinase | | 425 |
| 531 | PAS/PAC sensor-containing diguanylate cyclase | | 438 |
| 1486 | PAS/PAC sensor-containing diguanylate cyclase | | 515 |
| 4318 | PAS/PAC sensor-containing diguanylate cyclase | | 575 |
| 4352 | PAS/PAC sensor-containing diguanylate cyclase | | 631 |
| 706 | PAS/PAC sensor-containing diguanylate cyclase/phosphodiesterase | | 1267 |
| 1802 | PAS/PAC sensor-containing diguanylate cyclase/phosphodiesterase | | 839 |
| 2392 | PAS/PAC sensor-containing diguanylate cyclase/phosphodiesterase | | 567 |
| 4325 | PAS/PAC sensor-containing diguanylate cyclase/phosphodiesterase | | 679 |
| 4766 | PAS/PAC sensor-containing diguanylate cyclase/phosphodiesterase | | 958 |
| 306 | periplasmic sensor hybrid histidine kinase | | 776 |
| 2318 | periplasmic sensor hybrid histidine kinase | | 893 |
| 3996 | periplasmic sensor hybrid histidine kinase | | 656 |
| 4092 | periplasmic sensor hybrid histidine kinase | | 952 |
| 1856 | putative two-component response regulator | | 236 |
| 2525 | response regulator containing a CheY-like receiver domain and a GGDEF | | 364 |
| 3558 | response regulator containing a CheY-like receiver domain and an HD-GYP | | 332 |
| 3291 | response regulator receiver modulated CheB methylesterase | | 355 |
| 54 | response regulator receiver modulated diguanylate cyclase | | 313 |
| 2252 | response regulator receiver modulated diguanylate cyclase | | 313 |
| 2280 | response regulator receiver modulated diguanylate cyclase | | 437 |
| 2687 | response regulator receiver modulated diguanylate cyclase | | 417 |
| 614 | response regulator receiver modulated diguanylate cyclase/phosphodiesterase with PAS/PAC sensor | | 665 |
| 673 | response regulator receiver modulated diguanylate cyclase/phosphodiesterase with PAS/PAC sensor | | 755 |
| 1412 | response regulator receiver modulated diguanylate cyclase/phosphodiesterase with PAS/PAC sensor | | 873 |
| 3496 | response regulator receiver modulated diguanylate cyclase/phosphodiesterase with PAS/PAC sensor | | 681 |
| 3556 | response regulator receiver modulated diguanylate phosphodiesterase | | 130 |
| 1742 | response regulator receiver modulated metal dependent phosphohydrolase | | 352 |
| 2233 | response regulator receiver modulated metal dependent phosphohydrolase | | 704 |
| 3111 | response regulator receiver modulated metal dependent phosphohydrolase | | 361 |
| 3706 | response regulator receiver modulated metal dependent phosphohydrolase | | 365 |
| 3777 | response regulator receiver modulated metal dependent phosphohydrolase | | 358 |
| 92 | response regulator receiver protein | | 293 |
| 288 | response regulator receiver protein | | 296 |
| 603 | response regulator receiver protein | | 129 |
| 895 | response regulator receiver protein | | 390 |
| 1264 | response regulator receiver protein | | 120 |
| 1267 | response regulator receiver protein | | 124 |
| 1270 | response regulator receiver protein | | 563 |
| 1291 | response regulator receiver protein | | 145 |
| 1317 | response regulator receiver protein | | 322 |
| 2101 | response regulator receiver protein | | 449 |
| 2135 | response regulator receiver protein | | 124 |
| 2306 | response regulator receiver protein | | 362 |
| 2337 | response regulator receiver protein | | 354 |
| 2352 | response regulator receiver protein | | 131 |
| 2442 | response regulator receiver protein | | 398 |
| 3269 | response regulator receiver protein | | 260 |
| 3442 | response regulator receiver protein | | 576 |
| 4039 | response regulator receiver protein | | 408 |
| 4355 | response regulator receiver protein | | 310 |
| 566 | response regulator receiver sensor signal transduction histidine kinase | | 368 |
| 2338 | response regulator receiver sensor signal transduction histidine kinase | | 369 |
| 3888 | sensor histidine kinase response regulator | | 879 |
| 4056 | sensor histidine kinase response regulator | | 877 |
| 3303 | sensor histidine kinase, PAS domain-containing | | 538 |
| 4005 | Sensor protein fixL | | 886 |
| 602 | Sensor protein ZraS | | 341 |
| 1282 | sensor signal transduction histidine kinase | | 660 |
| 3446 | sensor signal transduction histidine kinase | | 476 |
| 3589 | sensor signal transduction histidine kinase | | 489 |
| 355 | sensor/response regulator hybrid | | 1376 |
| 3557 | sensor/response regulator hybrid | | 1425 |
| 93 | sensor/response regulator hybrid protein | | 1063 |
| 308 | sensor/response regulator hybrid protein | | 973 |
| 3709 | sensor/response regulator hybrid protein | | 1255 |
| 53 | sensor/response regulatory hybrid protein | | 1674 |
| 12 | Sensory/regulatory protein RpfC | | 642 |
| 3539 | Sigma-B modulator protein | | 271 |
| 3460 | signal transduction histidine kinase | | 111 |
| 3997 | signal transduction histidine kinase | | 826 |
| 1177 | signal transduction histidine kinase, nitrogen specific, NtrB | | 357 |
| 1885 | signal transduction protein | | 807 |
| 2092 | signal transduction response regulator | | 778 |
| 1702 | small GTP-binding protein | | 876 |
| 2033 | Transcriptional regulatory protein AfsQ1 | | 161 |
| 3304 | two component sigma54 specific Fis family transcriptional regulator | | 448 |
| 670 | two component system sensor histidine kinase, barA-like protein | | 1279 |
| 1857 | two component transcriptional regulator | | 251 |
| 3223 | two component transcriptional regulator | | 236 |
| 3285 | two component transcriptional regulator | | 247 |
| 3302 | two component transcriptional regulator | | 237 |
| 3445 | two component transcriptional regulator | | 244 |
| 3590 | two component transcriptional regulator | | 243 |
| 3640 | two component transcriptional regulator | | 242 |
| 1534 | two component, sigma54 specific, Fis family transcriptional regulator | | 459 |
| 3696 | two component, sigma54 specific, Fis family transcriptional regulator | | 475 |
| 4473 | two component, sigma54 specific, Fis family transcriptional regulator | | 499 |
| 55 | two-component hybrid sensor and regulator | | 381 |
| 3778 | two-component hybrid sensor and regulator | | 657 |
| 352 | two-component response regulator | | 153 |
| 1826 | Two-component response regulator with diguanylate cyclase | | 312 |
| 671 | two-component response regulator, modulated diguanylate cyclase | | 305 |
| 3641 | two-component sensor histidine kinase | | 653 |
| 354 | two-component system | | 335 |
| 1497 | Two-component system protein A | | 527 |
| 2307 | two-component system response regulator (hybrid family) protein | | 367 |
| 4344 | two-component system sensory histidine kinase | | 852 |
| 4004 | two-component system, chemotaxis family, CheB/CheR fusion protein | | 1646 |
